# Synthetic MRI for Radiotherapy Planning for Brain and Prostate Cancers: Phantom Validation and Patient Evaluation

**DOI:** 10.3389/fonc.2022.841761

**Published:** 2022-04-20

**Authors:** Pierrick Gouel, Sebastien Hapdey, Arthur Dumouchel, Isabelle Gardin, Eva Torfeh, Pauline Hinault, Pierre Vera, Sebastien Thureau, David Gensanne

**Affiliations:** ^1^Quantification en Imagerie Fonctionnelle-Laboratoire d’Informatique, du Traitement de l’Information et des Systèmes Equipe d’accueil 4108 (QuantIF-LITIS EA4108), University of Rouen, Rouen, France; ^2^Imaging Department, Henri Becquerel Cancer Center, Rouen, France; ^3^Radiotherapy Department, Henri Becquerel Cancer Center, Rouen, France

**Keywords:** synthetic MRI, radiotherapy planning, quantitative MRI, *T*_1_ mapping, *T*_2_ mapping

## Abstract

**Purpose:**

We aimed to evaluate the accuracy of *T*_1_ and *T*_2_ mappings derived from a multispectral pulse sequence (magnetic resonance image compilation, MAGiC^®^) on 1.5-T MRI and with conventional sequences [gradient echo with variable flip angle (GRE-VFA) and multi-echo spin echo (ME-SE)] compared to the reference values for the purpose of radiotherapy treatment planning.

**Methods:**

The accuracy of *T*_1_ and *T*_2_ measurements was evaluated with 2 coils [head and neck unit (HNU) and BODY coils] on phantoms using descriptive statistics and Bland–Altman analysis. The reproducibility and repeatability of *T*_1_ and *T*_2_ measurements were performed on 15 sessions with the HNU coil. The *T*_1_ and *T*_2_ synthetic sequences obtained by both methods were evaluated according to quality assurance (QA) requirements for radiotherapy. *T*_1_ and *T*_2_ *in vivo* measurements of the brain or prostate tissues of two groups of five subjects were also compared.

**Results:**

The phantom results showed good agreement (mean bias, 8.4%) between the two measurement methods for *T*_1_ values between 490 and 2,385 ms and *T*_2_ values between 25 and 400 ms. MAGiC^®^ gave discordant results for *T*_1_ values below 220 ms (bias with the reference values, from 38% to 1,620%). *T*_2_ measurements were accurately estimated below 400 ms (mean bias, 8.5%) by both methods. The QA assessments are in agreement with the recommendations of imaging for contouring purposes for radiotherapy planning. On patient data of the brain and prostate, the measurements of *T*_1_ and *T*_2_ by the two quantitative MRI (qMRI) methods were comparable (max difference, <7%).

**Conclusion:**

This study shows that the accuracy, reproducibility, and repeatability of the multispectral pulse sequence (MAGiC^®^) were compatible with its use for radiotherapy treatment planning in a range of values corresponding to soft tissues. Even validated for brain imaging, MAGiC^®^ could potentially be used for prostate qMRI.

## 1 Introduction

MRI displays contrast that is principally dependent on 3 intrinsic parameters: *T*_1_, *T*_2_, and *ρ*. For diagnostic purposes, weighted sequences aim to obtain a contrast depending on the nature of the tissue studied by highlighting one of these 3 parameters. The drawback of such an approach is the complex nature of the signal. It differs from one manufacturer to another for the same types of weighted images, and it is not possible to extract the quantitative values of intrinsic parameters.

Meanwhile, quantitative magnetic resonance imaging (qMRI) (1) has been proposed for many years to obtain a mapping of the measurement of one of the intrinsic parameters. These sequences are based on a known mathematical expression of the signal, depending on only one of the 3 intrinsic parameters, to calculate the intrinsic parameter at the voxel level by solving the signal expression. qMRI has not been widely used because of the prohibitive time required for image acquisition, measurement uncertainties (2), and diagnostic success of weighted sequences.

qMRI allows the characterization of tissues and pathologies for a wide range of diagnostic applications (1, 3). It can provide quantitative *T*_1_ and *T*_2_ tissue mappings at high spatial resolution (4), which can be useful for radiotherapy to differentiate recurrent tumors from benign tissues (5), to improve contouring (6) and optimize treatment planning (7), or to detect early effects of irradiation (8).

Another application of qMRI after the acquisition of *T*_1_ or *T*_2_ mapping is in mathematically generating synthetic *T*_1_- or *T*_2_-weighted sequences (synMRI). This technique allows the operator to modify the repetition time (TR) and echo time (TE) values in order to obtain a multi-contrast MRI signal from a single acquisition. The main advantages of this technique are the reduction of the scan time and improvement of patient throughput. Evaluations of this new technique are essentially based on brain studies (9). Several authors have also proposed synMRI for the study of the knee (10, 11), lumbar intervertebral disc degeneration (12), and prostate cancer (13), but always with the acquisition parameters for diagnostic purposes favoring the signal-to-noise ratio (SNR).

The first quantitative methods proposed in the literature were inspired by the methods used in nuclear magnetic resonance (NMR) (1). For *T*_1_ measurement, the reference technique is an inversion recovery (IR) sequence that remains too time-consuming for routine use. To reduce the acquisition time, a variable flip angle (VFA) method has been proposed (14). This method allows a rapid return to equilibrium magnetization of the repeated sequences with low tilt angles of longitudinal magnetization. At the end of each sequence, the signal is measured after the application of a gradient echo (GRE). The homogeneity of the *B*_1_ field must be checked and corrected when necessary (14). For *T*_2_ measurement, the reference sequence is based on a single spin echo (SE) sequence with signal recording after each multi-echo (ME-SE) (1).

The previous methods allow mapping of only one intrinsic parameter (*T*_1_ or *T*_2_), but not all three (*T*_1_, *T*_2_, and *ρ*). In recent years, several authors have proposed multispectral pulse sequences that enable recording the signal several times in a single sequence in order to calculate the 3 intrinsic parameters while limiting the acquisition time. The characteristics are then mapped, and each voxel represents the value of *T*_1_, *T*_2_, or *ρ*. Warntjes et al. (15) proposed a quicker method based on a multi-parametric pulse sequence (QRAPMASTER) from a ME saturation recovery acquisition using a turbo spin echo (TSE) readout to generate *T*_1_, *T*_2_, and *ρ* mappings applied to brain imaging. At the same time, measurement of the *B*_1_ field is performed to correct the inhomogeneity of the amplitude radiofrequency (RF) emission. Inspired by the QRAPMASTER sequence, the magnetic resonance image compilation (MAGiC^®^) sequence, available on General Electric Healthcare systems (GE Healthcare, Milwaukee, WI, USA), has been made commercially available (16).

Several publications evaluated the performance of MAGiC^®^ compared to conventional sequences (IR and ME-SE) with a phantom on a 3-T MRI, with discordant results (10, 17, 18). Although the SNR is lower than that at 3 T (19), MRI at 1.5 T has advantages, such as the reduction of artifacts and a better homogeneity of the radiofrequency field, which has a direct impact on the qMRI image quality and which are of major interest for the purpose of anatomical segmentation radiotherapy planning. Furthermore, Guarnaschelli et al. showed that 3-T MRI may reveal a significantly smaller tumor volume for high-grade gliomas and that target volume segmentation for radiation treatment may be better at 1.5 *T* (20). West et al. (21) studied the similarities and differences of the QRAPMASTER^®^ sequence at 1.5- and 3-T field strengths in brain tissue segmentations. This study showed that most of the different brain tissues were classified identically at both field strengths, although some regional differences were observed, such as variations in the segmented tissue volumes. They also noticed a better repeatability of measurements at 1.5 T.

To our knowledge, no study has evaluated the quality of MAGiC^®^ measurements at 1.5 T compared to conventional qMRI measurement methods to obtain multi-contrast synthetic sequences applied to anatomic segmentation for radiotherapy treatment planning, given its mandatory quality assurance (QA) requirements (7, 22). In this work, we evaluated the accuracy, the range of use, and the reproducibility of *T*_1_ (MAGiC^®^ and GRE-VFA) and *T*_2_ (MAGiC^®^ and ME-SE) based on phantom studies. Performance was evaluated with two coils, a head and neck unit (HNU) coil and a BODY coil with the acquisition parameters for radiotherapy treatment. We also compared the *in vivo T*_1_ and *T*_2_ measurements in human brain and prostate tissues for radiotherapy application.

## 2 Materials and Methods

### 2.1 Phantoms

**Figure 1:**
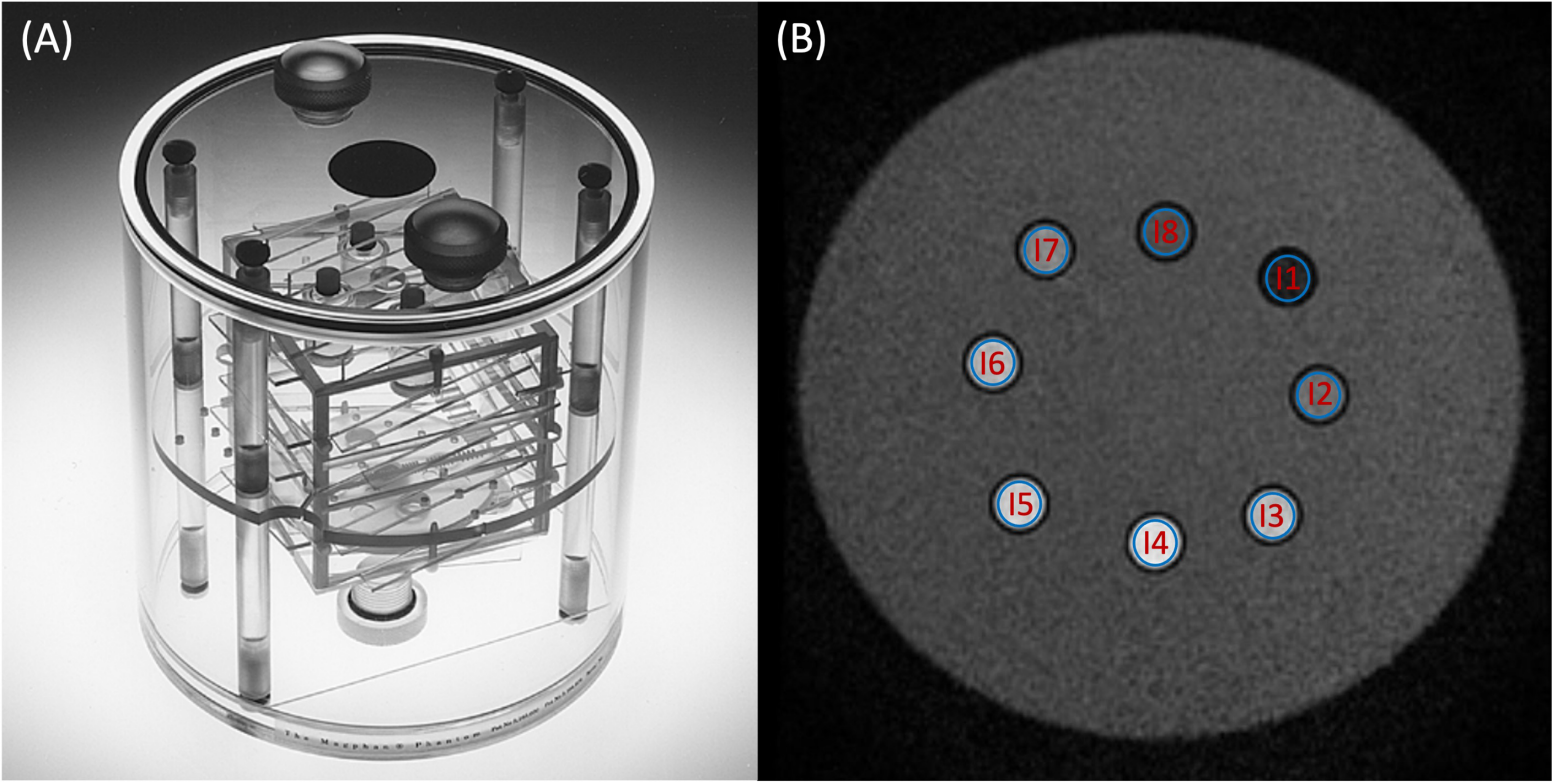
**(A)** Magphan^®^ SMR170 (The Phantom Laboratory, Salem, NY, USA). **(B)** Synthetic *T*_2_-weighted image of the homemade phantom used in this study. The *blue* volumes of interest (VOIs) **(B)** represent the different inserts measured in this study.

The Magphan**^®^** SMR170 (The Phantom Laboratory, Salem, NY, USA) was used (**Figure 1A**). It has conventional cylindrical housing for test groups and a removable end plate for internal access. The acrylic cylinder has an outer diameter of 20 cm and an inner diameter of 19 cm. It contains features that allow comprehensive testing of achievable MRI scanner QA parameters (QAp). Geometric distortion, spatial linearity, pixel size, slice increment, slice thickness, high-contrast detectability, low-contrast detectability, and uniformity were measured. These parameters have already been described in previous publications (7, 22–24).

To cover a wider range of *T*_1_ and *T*_2_ values, a homemade phantom was developed (**Figure 1B**). It is a box composed of a 0.3-cm-thick plastic wall filled with water. It is composed of eight inserts, 5 cm high and 1 cm wide. Each sample contained a solution of 1.5 ml of gadoteric acid (DOTAREM^®^, 0.5 mmol/ml). The concentrations and the relaxation times (*T*_1_ and *T*_2_) were, respectively, for insert 1 (I1) = 8 × 10^−3^ mmol/ml (29.1 and 24.7 ms), I2 = 4 × 10^−3^ mmol/ml (57.7 and 48.8 ms), I3 = 2 × 10^−3^ mmol/ml (113.1 and 95.5 ms), I4 = 1.10^−3^ mmol/ml (217.8 and 181.7 ms), I5 = 4 × 10^−4^ mmol/ml (489.7 and 399.6 ms), I6 = 2 × 10^−4^ mmol/ml (838.9 and 665.6 ms), I7 = 1 × 10^−4^ mmol/ml (1,303.8 and 997.5 ms), and I8 = 0 mmol/ml (2,385.7 and 1,311.3 ms). The effective concentrations of each insert were verified by an MRI spectrometer (Biospec 47/40 imager; Bruker, Billerica, MA, USA) under clinical conditions at 1.5 T to define the values of the *T*_1_ and *T*_2_ reference relaxation times for a temperature of 22°C after the end of the measurement phantom study.

### 2.2 *T*_1_ and *T*_2_ Measurements

#### 2.2.1 Phantom Data Acquisitions

All acquisitions were performed on a 1.5-T Optima MR450w clinical MRI (GE, software version DV26.0-R01) at a temperature of 22°C (±0.5°C) maintained by a continuous air conditioning system. The sequences were acquired on the phantoms in 2D axial orientation according to the acquisition parameters given in **Table 1**. It consisted of the MAGiC^®^ sequence for generating *T*_1_, *T*_2_, and *B*_1_ mappings (16) and conventional sequences. The conventional methods used were four GRE-VFA with a destructive gradient of the residual magnetization between each change of the tilt angle (14) for the measurement of *T*_1_ and an ME-SE sequence with eight echoes for the measurement of *T*_2_ (1). To compare the performance of the methods, the acquisition parameters of the MAGiC^®^ sequence were similar to those of the conventional sequences (**Table 1**). Two coils were used for the qMRI evaluation: the HNU coil and the BODY coil in 24AA2 configuration (combination of coils anterior and posterior). Due to its size, the Magphan^®^ SMR170 phantom could not be imaged with the HNU coil for the synMRI QA analysis.

**Table 1:** Acquisition parameters of the phantom images.

Sequences	MAGiC^®^	GRE-VFA (*T*_1_)	ME-SE (*T*_2_)
No. of TE	2	1	8
TE values (ms)	21.8–87.1	2.2	8.1–16.2–24.3–32.2–40.3–48.4–56.4–64.5
TR values (ms)	4,000	8	1,000
Bandwidth (kHz)	31.25	31.25	31.25
Slice thickness (mm)	2.5	2.5	2.5
Flip angle (deg)	120–180–90	3–10–20–30	90
Pixel size (mm)	1.02 × 1.02	1.02 × 1.02	1.02 × 1.02

TE, echo time; TR, repetition time.

#### 2.2.2 qMRI Measurements From the MAGiC^®^ Sequence

*T*_1_ and *T*_2_ mappings from the MAGiC**^®^** sequence were calculated using SyMRI**^®^** software (version 100.1.1; SyntheticMR AB, Linköping, Sweden) available on the MRI acquisition station. The expression of the signal, *S*_MAGiC_, is given in Equation 1.


$$S_{\text{MAGiC}}=K_{\text{GE}}\cdot \rho \cdot e^{-\text{TE}/T2}\cdot \frac{1-[1-\cos(B1\theta )]\cdot \text{exp}(-\text{TI}/T1)-\cos(B1\theta )\cdot \exp(-\text{TR}/T1)}{1-\cos(B1\alpha )-\cos(B1\theta )\cdot \text{exp}(-\text{TR}/T1)}\;(1)$$

where *K*_GE_ is a global intensity scaling factor taking into account coil sensitivity, RF chain amplification, and voxel volume that is specific to MRI. *ρ* is the proton density, *B*_1_ is the radiofrequency field, TI is the macroscopic magnetization reversal time, TR is the repetition time, and TE is the echo time.

The algorithm for calculating *T*_1_ and *T*_2_ mappings is based on a least-squares adjustment of the signal intensity for each voxel. The volumes of interest (VOIs) of 200 voxels were positioned on the homemade phantom on each sample at slice 13/26. The mean *T*_1_ and *T*_2_ values, as well as the standard deviation (SD), were calculated for each VOI.

#### 2.2.3 qMRI Measurements From Conventional Sequences

For *T*_1_ measurements using the GRE-VFA sequence, the expression of the signal is given in Equation 2.


$$S_{T1}=K_{\text{GE}}\cdot \rho \cdot \sin(\theta )\cdot \frac{1-e\left(-\frac{\text{TR}}{T1}\right)\cdot e(-\text{TE}/T2*)}{1-\cos(\theta )\;x\;\exp(-\text{TR}/T1)}\;(2)$$

The *B*_1_ field uniformity correction using the Bloch–Siegert offset method (25) was evaluated on phantom once a week for 6 weeks for both coils. The mean values were calculated; the obtained calibration curves are given as supplementary data (**Supplementary Figures S1, S2**). Since the systematic errors for both coils were between −1.6% and 1.8% whatever the coil used, no correction of the *B*_1_ field uniformity was applied to the data obtained with the *T*_1_ mappings generated from the conventional sequences on phantoms and on patients.

For the *T*_2_ measurement, the signal expression verified Equation 3.


$$S_{T2}=K_{\text{GE}}\cdot \rho \cdot 1-e^{-\text{TR}/T1}\cdot e^{-\text{TE}/T2}\;(3)$$

*T*_1_ and *T*_2_ mappings were calculated using the OleaNova+^®^ module of Olea Sphere^®^ software (version 3.0; OLEA MEDICAL, La Ciotat, France) by solving Equations 2 (*T*_1_) and 3 (*T*_2_). The VOIs of 180 voxels on *T*_1_ and *T*_2_ mappings were defined on the homemade phantom on each sample at slice 13/26. The mean *T*_1_ and *T*_2_ values, as well as the SD, were calculated for each VOI.

#### 2.2.4 synMRI Measurements

From the quantitative maps obtained, *T*_1_- and *T*_2_-weighted images were synthesized from these maps with the syMRI**^®^** software for the MAGiC**^®^** sequence and OleaNova+**^®^** for the conventional sequences. For both methods, values of TE = 25 ms and TR = 600 ms were defined for the *T*_1_-weighted synthetic sequence and TE = 65 ms and TR = 1,900 ms for the *T*_2_-weighted synthetic sequence. All sequences were exported and saved in Artiscan**^®^** software (version 4.1.18; AQUILAB, Loos, France) for QA analysis. For each sequence, the 2D geometric distortion, spatial linearity, pixel size, slice increment, slice thickness, high-contrast detectability, low-contrast detectability, and uniformity were measured (24, 26).

#### 2.2.5 *In Vivo* Measurements

After giving their free and informed consent (Institutional Review Board no. 2103B), 10 patients (2 women and 8 men) with oncologic diseases accepted additional acquisitions with MAGIC**^®^** and conventional sequences. Five patients imaged on the brain (mean age = 56 years, range = 39–76 years) had indications of glioblastoma, and five patients imaged on the pelvis (mean age = 67 years, range = 45–80 years) had indications of prostate cancer. The same acquisition parameters were used (**Table 1**), except for the pixel size, which was increased to 2 × 2 mm for both methods to make the acquisition time acceptable (5 min, 36 s for MAGiC**^®^** and 2 min, 36 s GRE-VFA + 3 min, 38 s for ME-SE). *T*_1_ and *T*_2_ mappings were calculated from the MAGiC**^®^** sequence and conventional sequences using the same procedures as in the phantom experiments. The VOIs measured for both patients on *T*_1_ and *T*_2_ mappings corresponded to the theoretical range of application of the MAGiC**^®^** sequence. For the brain and prostate, 3 VOIs (oval and identical of 44 voxels and size 2 × 2 × 2.5 mm) were manually positioned on the same anatomical and non-pathological areas at the identical slice number for each *T*_1_ and *T*_2_ mapping obtained with both methods. For the brain, the anatomical areas were white matter (WM), gray matter (GM), and cerebrospinal fluid (CSF). For 1 of the 5 patients, a VOI was positioned over the known calcium lesion. For the pelvis, anatomical areas of the prostate, gluteal muscle, and subcutaneous fat of the buttocks were studied.

### 2.3 Statistical Analysis

A descriptive statistic was carried out from the *T*_1_ and *T*_2_ maps obtained using the phantom at 1.5 T. This analysis focused on the calculation of the mean and relative percentage difference between the measured and the reference value of the VOIs positioned on the image of each insert. The standard deviation and coefficient of variation (CV) (27) representing the voxel-to-voxel measurement uncertainty were given as percentages in each insert for all measurement methods and for each coil. The agreement of the mean values of the *T*_1_ and *T*_2_ maps was compared to the reference values calculated at 1.5 T using a Bland–Altman graph (28) for each coil configuration. The mean relative difference of 14 repeated measurements (in percent) with respect to the first measurement was calculated and plotted in boxplot graphs (29). Repeatability was quantified with the CV within the VOIs over the 15 scan sessions, and the 95% confidence interval of the CV was calculated. A comparison was performed on the *T*_1_ and *T*_2_ maps obtained on the brain and prostate between the two measurement methods using a Bland–Altman graph (28) with the 95% limits of agreement as the mean difference (a low limit represents the mean − 1.96 × SD and a high limit represents the mean + 1.96 × SD). The mean VOIs measured with the minimum and maximum values of the different anatomical areas, as well as the average of the relative differences with the minimum and maximum values, were given for analysis.

All statistical analyses were performed using Python^®^ software (version 3.7.0).

## 3 Results

### 3.1 Phantom Measurements

#### 3.1.1 Accuracy and Range of *T*_1_ and *T*_2_ Measurements

**Figure 2:**
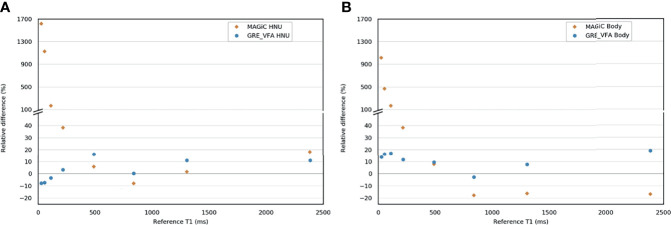
Bland–Altman graph representing the relative difference (in percent) between the *T*_1_ measurement with the MAGIC^®^ and the gradient echo with variable flip angle (GRE-VFA) sequences for the head and neck unit (HNU) coil **(A)** and the BODY coil **(B)** compared to the reference measurements.

Regarding the *T*_1_ measurement, **Figure 2** and **Supplementary Tables S1, S2** show that the GRE-VFA sequence with the HNU coil provided a relatively accurate estimation of *T*_1_ (relative differences ranged from −7.9% to +16.0%). Larger biases were measured with the BODY coil, which systematically gave lower measurements with maximum errors from −2.8% to +18.8%. The MAGIC^®^ sequence gave *T*_1_ measurements consistent with the expected values, with biases less than 20% in the range 490–2,386 ms. On the other hand, for *T*_1_ ≤218 ms, the measurements were highly overestimated (see outliers from 113 ms and below in **Figure 2**). The HNU coil gave better results than the BODY coil in the *T*_1_ measurement from 490 to 2,386 ms.

**Figure 3:**
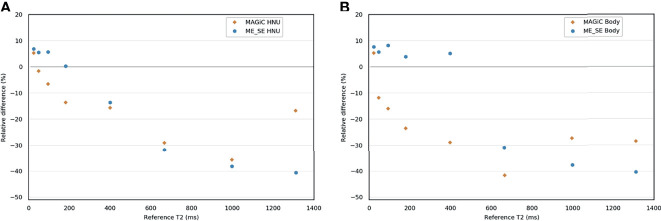
Bland–Altman graph representing the relative difference (in percent) between the *T*_2_ measurements with the MAGIC^®^ and the multi-echo spin echo (ME-SE) sequences for the head and neck unit (HNU) coil **(A)** and the BODY coil **(B)** compared to the reference measurements.

For the ME-SE sequence, between 25 and 400 ms, the HNU coil provided accurate *T*_2_ measurements (relative differences ranged from −13.6% to +6.8%) (**Figure 3** and **Supplementary Tables S1, S2**). For higher *T*_2_ (>400 ms), the measurement was systematically underestimated, with maximum errors from −86.1% to −31.8%. With the BODY coil, the ME-SE sequence also gave good agreement for *T*_2_ measurements ≤400 ms (relative differences ranged from +3.9% to +8.2%). The MAGIC^®^ sequence gave *T*_2_ measurements with a bias less than 20% for *T*_2_ ≤400 ms (relative differences ≤15.7%) with the HNU coil. With the BODY coil, the MAGiC^®^ sequence was less efficient, with biases lower than 20% on *T*_2_ values lower than 95.2 ms.

**Figure 4:**
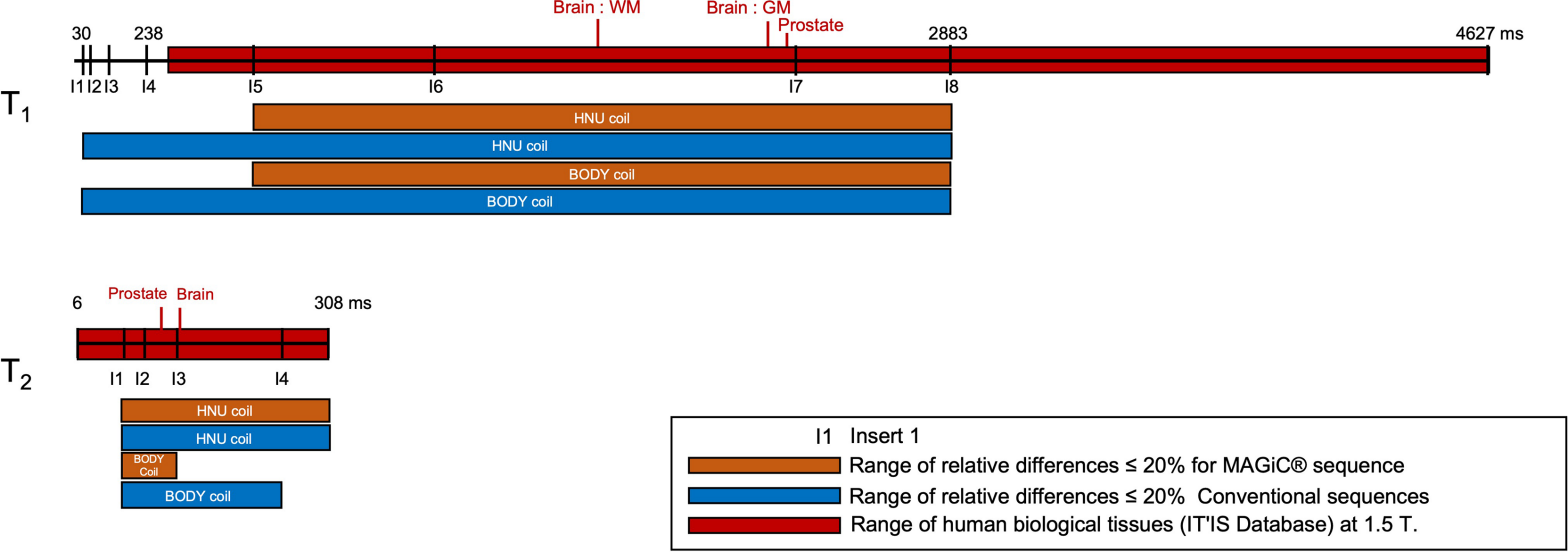
Comparisons of the results of the relative differences ≤10% (except inserts 5, 7, and 8, where the results were slightly higher and given on the diagram) of the *T*_1_ and *T*_2_ mappings for the MAGiC^®^ method (*thick orange line*) and the conventional method (*thick blue line*) for the range of values measured on phantom compared to the reference values (*dotted line mark*), to the range of human biological tissues (30) at 1.5 T (*thick red line mark*), and to the range of human biological tissues of interest for radiotherapy planning at 1.5 T (*green line mark*).

The known ranges of relaxation times for human biological tissues at 1.5 T are listed in a database from various articles published in the literature (30) and presented in **Figure 4**. This figure shows that, in our study, the *T*_1_ and *T*_2_ values estimated with the conventional and MAGiC^®^ methods with error less than 20% covered a wide range of known relaxation times of human biological tissues at 1.5 T. In particular, those of interest for radiotherapy treatment planning are between 30 and 2,883 ms and between 25 and 182 ms for *T*_1_ and *T*_2_ measurements, respectively. The MAGiC^®^ method showed higher biases than those of the conventional method for *T*_1_ values <490 ms and for *T*_2_ values >95 ms.

#### 3.1.2 Reproducibility and Repeatability of *T*_1_ and *T*_2_ Measurements

**Figure 5:**
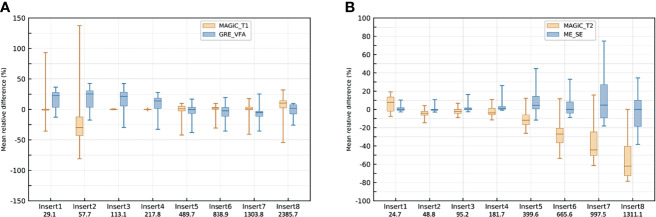
Box plot of the repeated measurements (*n* = 14) with the head and neck unit (HNU) coil of *T*_1_ mappings for the MAGiC^®^ sequence and gradient echo with variable flip angle (GRE-VFA) **(A)** and *T*_2_ mappings for the MAGiC^®^ sequence and multi-echo spin echo (ME-SE) **(B)**. The values of the relative differences (in percent) are given with the associated medians, first and third quartiles, and the maximum and minimum values.

**Figures 5A** **, B** give the evolution of the *T*_1_ and *T*_2_ measurements over time for each insert for the conventional and MAGiC^®^ methods using the HNU coil. The relative difference with the associated median, the first and third quartiles, and the minimum and maximum values are given between the first measurement and each of the 14 measurements repeated over 5 months. This figure shows that *T*_1_ measurement with the MAGIC^®^ sequence was stable over 5 months, with a median close to zero for all inserts, except for insert 2 (median error greater than −25%), and a dispersion between ±10%, but with some extreme measurements. On the other hand, *T*_1_ measurements with the GRE-VFA method were less reproducible for the first four inserts. The *T*_2_ measurement of the inserts was globally reproducible over 5 months with the ME-SE method, with a median error less than 6% compared to the MAGiC^®^ method, which showed worse results, especially for the last four samples (medians errors between −13% and −63%). The dispersion and extreme values of the relative differences of each sample increased as the *T*_2_ values increased for both methods.

**Figure 6:**
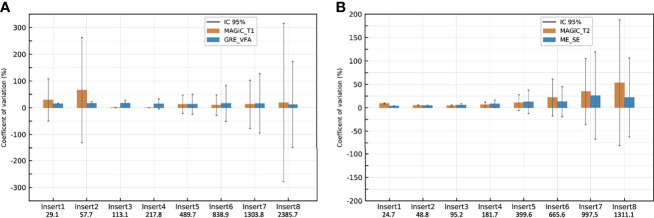
Coefficients of variation of the repeated measurements (*n* = 15) with the head and neck unit (HNU) coil of *T*_1_ mappings for MAGiC ^®^and gradient echo with variable flip angle (GRE-VFA) **(A)** and *T*_2_ mapping for MAGiC^®^ and multi-echo spin echo (ME-SE) **(B)**. *Vertical lines* show the 95% confidence intervals.

**Figures 6A** **, B** give the CVs of repeated measurements (*n* = 15) over 5 months with the HNU coil of *T*_1_ mapping for MAGiC ^®^and GRE-VFA and *T*_2_ mapping for MAGiC^®^ and ME-SE, with the associated 95% confidence intervals. The figure shows that the CVs were elevated for *T*_1_ measurement with the MAGiC^®^ method for the first two inserts (29% and 66%). For the other inserts, all CVs were similar and lower than 16% for both measurement methods. The CVs were similar and comparable for the *T*_2_ measurement for both measurement methods. The CV values were less than 10% for the first four inserts. Then, they degraded for the last four inserts with a maximum of 34% for MAGiC^®^, whose CV values were higher than those of ME-SE for the last three inserts. An increase in the CV confidence interval for both *T*_1_ and *T*_2_ measurement methods was observed with higher relaxation times studied.

#### 3.1.3 QA Analysis on Synthetic *T*_1_- and *T*_2_-Weighted Images

Synthetic *T*_1_- and *T*_2_-weighted images were obtained with the Magphan^®^ SMR170 phantom. These images were synthesized from the *T*_1_ and *T*_2_ mappings with the syMRI^®^ software for the MAGiC^®^ sequence and OleaNova+^®^ for the conventional sequences with the same TE and TR values. For each sequence, the geometric distortion, spatial linearity, pixel size, slice increment, slice thickness, high-contrast detectability, low-contrast detectability, and uniformity were measured and are shown in **Table 2**. The expected optimal values of each parameter were given for comparison to the measurements of each sequence obtained from *T*_1_ and *T*_2_.

**Table 2:** Values of the quality assurance (QA) parameters for phantom.

		**Optimal values**	**MAGiC^®^ *T*_1_**	**GRE-VFA**	**MAGiC^®^ *T*_2_**	**ME-SE**
Geometric distorsion	Mean diameter (mm)	190.00	190.38	189.47	190.11	189.47
	%	0.00	0.04	0.14	0.37	0.14
Spatial linearity	(%)	0.00	−0.13	−1.46	−0.16	−1.51
Pixel size	*x* (mm)	1.02	1.00	1.02	1.00	1.02
	*y* (mm)	1.02	1.02	1.02	1.02	1.01
Slice increment	(mm)	2.5	2.47	2.24	2.47	2.26
Slice thickness	(mm)	2.5	2.22	2.86	2.37	2.9
High-contrast detectability	Resolution (pl/cm)	Minimal value	6.95	6.77	7.55	6.80
Low-contrast detectability	SNR	Maximal value	58.64	267.02	79.29	353.94
	Noise (%)	0.00	3.73	5.98	5.52	5.41
Uniformity	(%)	100.00	92.08	89.30	89.34	89.35

GRE-VFA, gradient echo with variable flip angle; ME-SE, multi-echo spin echo; SNR, signal-to-noise ratio.

The results showed a distortion lower than 0.5% for both *T*_1_ and *T*_2_ measurement methods. The measurement of spatial linearity showed good results (<1.5%), but was better for the MAGiC^®^ measurement method for *T*_1_ and *T*_2_. Pixel size was comparable for both *T*_1_ and *T*_2_ measurement methods. The slice thickness was slightly below the expected optimal value for the MAGiC^®^ method (between −0.22 and −0.13 mm), while it was higher for the conventional method in *T*_1_ and *T*_2_ (+0.4 mm). The high-contrast detectability was comparable for both measurement methods in *T*_1_ and worse for the MAGiC^®^ method (7.55 pl/cm) compared to the conventional method (6.8 pl/cm). The conventional method showed better low-contrast detectability in *T*_1_ and *T*_2_ compared to the MAGiC^®^ method with a similar noise level. The measured uniformity was good for both methods in *T*_1_ and *T*_2_ (>89%).

### 3.2 *In Vivo* Measurements

**Figure 7:**
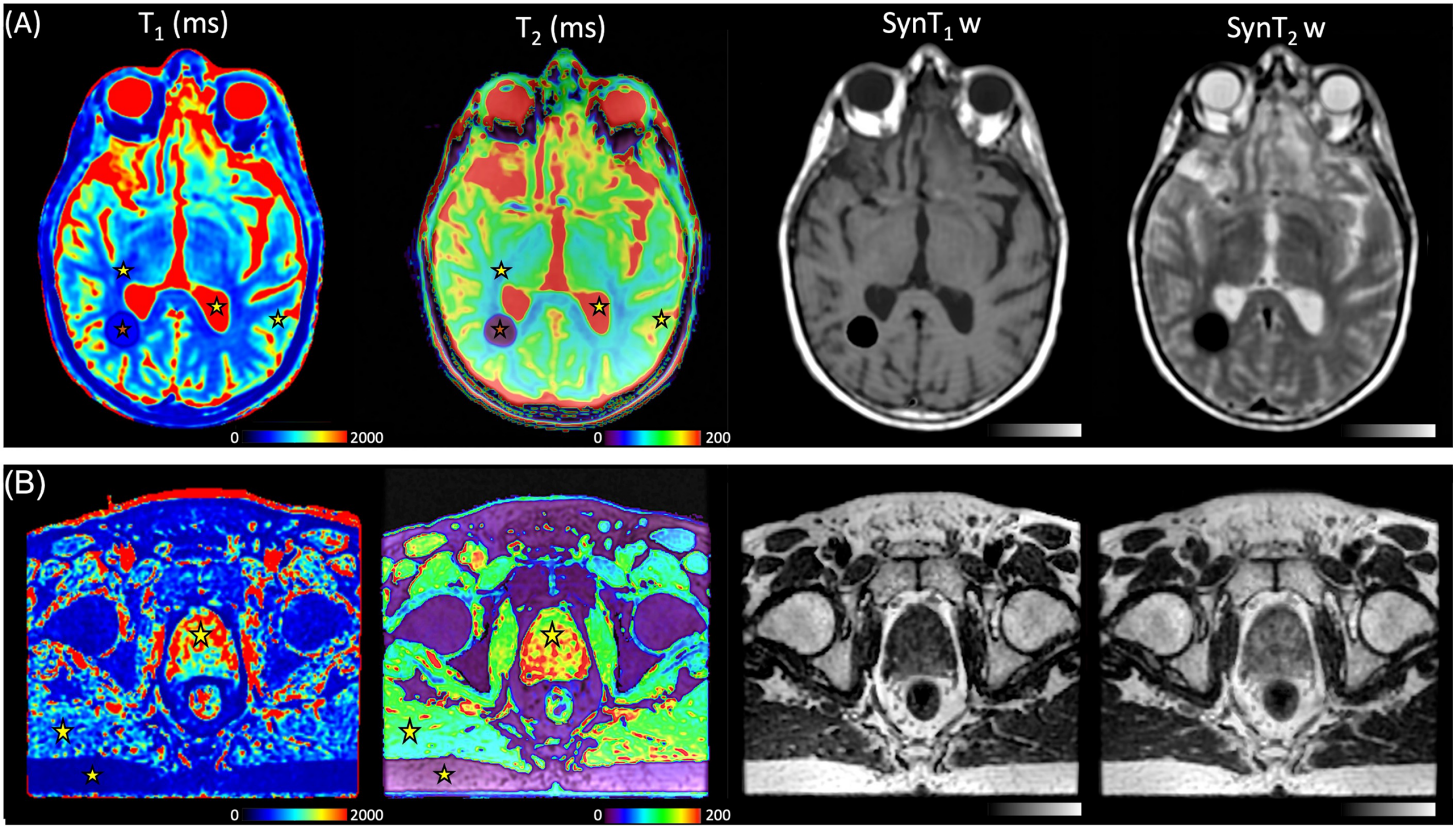
**(A)** *T*_1_ maps, *T*_2_ maps, SynT_1_w, and SynT_2_w of the brain in a 39-year-old man with known calcified lesion. *Stars* represent the volume of interest (VOI) of the mean measured values, white matter (WM), gray matter (GM), and cerebrospinal fluid (CSF), for the MAGiC^®^ method. **(B)** *T*_1_ maps, *T*_2_ maps, SynT_1_w, and SynT_2_w of the pelvis in a 72-year-old man with prostate cancer. *Stars* represent the VOI of the mean measured fat, muscle, and prostate for the MAGiC^®^ method and conventional sequences.

**Figure 7** shows an example of a synthetic image obtained from the MAGiC^®^ sequence for the study of the brain and prostate. The images offered a level of visual image quality that allows the study of the different tissues for radiotherapy treatment planning.

#### 3.2.1 Comparison of *T*_1_ and *T*_2_ Measurements in the Human Brain

**Figure 8:**
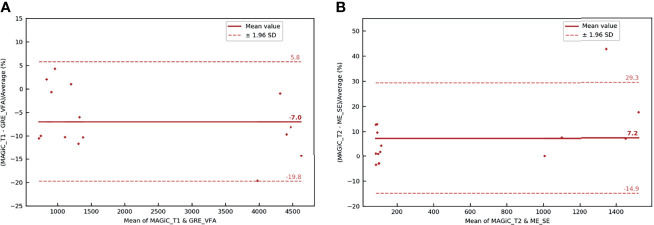
Bland–Altman graph representing the relative difference (in percent) between the *T*_1_ **(A)** and *T*_2_ **(B)** maps with the MAGIC^®^ method and conventional sequences for the five patients scanned on the brain with the head and neck unit (HNU) coil. The *red solid line* marks the mean of the difference; the *red dotted lines* mark the mean ± 1.96 standard deviation of the difference.

In **Figures 8A, B**, the overall results showed that the average differences between the two measurement methods for the different anatomical areas of the brain studied were −7% for the *T*_1_ measurement and 7.2% for the *T*_2_ measurement. All measurements were within the limits of agreement, except for one extreme measurement for a high *T*_2_ value (>30%) and one measurement at the limit of agreement for a high *T*_1_ value (−19.8%). The results showed that the two measurement methods are comparable for obtaining *T*_1_ and *T*_2_ mappings. In **Figure 7A**, a brown star represents the measurement of a calcium lesion found in 1 of the 5 patients. We showed a significant difference in the *T*_1_ measurement with a relative difference of +69.6% between the MAGiC^®^ and GRE-VFA methods, whereas the *T*_2_ measurement was comparable for both methods (22 ms for MAGiC^®^ *versus* 21 ms for ME-SE).

#### 3.2.2 Comparison of *T*_1_ and *T*_2_ Measurements in the Human Prostate

**Figure 9:**
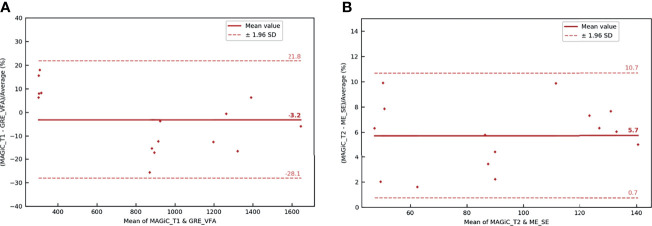
Bland–Altman graph representing the relative difference (in percent) between the *T*_1_ **(A)** and *T*_2_ **(B)** maps with the MAGIC^®^ method and conventional sequences for the five patients scanned on the prostate with the BODY coil. The *red solid line* marks the mean of the difference; the *red dotted lines* mark the mean ± 1.96 standard deviation of the difference.

The overall results (**Figures 9A, B**) showed that the average differences between the two measurement methods for the different anatomical areas of the prostate studied were −3.2% for the *T*_1_ measurement and 5.7% for the *T*_2_ measurement. All measurements were within the limits of agreement. The results showed that the two measurement methods were similar for obtaining *T*_1_ and *T*_2_ mappings.

## 4 Discussion

Our study compared, at 1.5 T, the performance of *T*_1_ and *T*_2_ measurements using a combined method enabling a single acquisition to obtain *T*_1_ and *T*_2_ mappings (MAGiC^®^) and conventional sequences (GRE-VFA and ME-SE) with acquisition parameters adapted to radiotherapy planning. The results of the homemade phantom showed good agreement between the two measurement methods with respect to the reference values for a wide range of *T*_1_ values, compatible with the biological tissues encountered in radiotherapy. The QAp evaluated on the *T*_1_ and *T*_2_ synthetic weighted images for both measurement methods are in agreement with the recommendations of imaging for contouring purposes for radiotherapy planning (7, 22), with a very low average distortion (<1%) and spatial linearity (<1.5%) and an excellent uniformity (>89%). For both brain and prostate tissues, the estimations by the two qMRI methods of *T*_1_ and *T*_2_ mappings were comparable.

To our knowledge, this is the first study evaluating the feasibility of the MAGiC^®^ method for radiotherapy imaging of the male pelvis and, in particular, the prostate (**Figure 6B**). However, these results obtained on 5 patients need to be further evaluated on a larger number of patients.

For *T*_1_ measurement, **Figure 2** demonstrates the good agreement between the reference values and the GRE-VFA sequence for the whole range of measurements (relative error, <18.8%), with a reproducibility close to 20% over 5 months for both coils. The MAGiC^®^ sequence gave discordant values (>20%) for low *T*_1_ values, below 218 ms. A more detailed analysis between 200 and 500 ms would refine this analysis, but caution should be taken when using this sequence for the study of low *T*_1_ values. This issue was also found for the calcified lesion in one patient in the brain group (see brown star in **Figure 7A**), where the *T*_1_ values measured by the MAGiC^®^ method were 500 and 152 ms for GRE-VFA. However, it was not found for the measurement of subcutaneous fat in the patients of the prostate group (**Supplementary Table S3**), with average measurement differences between the MAGiC^®^ method and EG-VFA ranging from 6% to 15%. Our results are in agreement with those of Li et al. (17) at 3 T, who found comparable results between the 2 methods in the range of *T*_1_ values they studied (For *T*_1_ values measured above 1131 ms). Our results are also in agreement with the results of Kim et al. (18), showing that, at 3 T, the 2 methods gave comparable *T*_1_ measurements below 433 ms. For *T*_1_ values measured between 1,131 and 2,117 ms and *T*_2_ values between 59 and 230 ms. This study showed relative differences between −8% and 25% for *T*_1_ and −27% and +10% for *T*_2_ compared to the phantom reference values. Kim et al. (18) also compared MAGiC^®^ with conventional sequences (SE multi-TR for *T*_1_ measurement and ME-SE for *T*_2_ measurement) over a range of measured *T*_1_ values from 70 to 2,875 ms and *T*_2_ values from 51 to 1,437 ms. The results showed that the *T*_1_ measurements were comparable for both methods over a range of values between 433 and 2,875 ms and were significantly different between the two methods for *T*_1_ values measured between 70 and 382 ms and over the range of *T*_2_ values. Lee et al. (10), whose phantom evaluation of *T*_2_ measurements performed with the MAGiC^®^ sequence in a value range between 30 and 110 ms, showed very good linear regression (*y* = 1.022*x* + 0.9903, *R^2^* = 0.9985) compared to the ME-SE sequence (*B*_0_ = 3 T).

The accuracy and discrepancy between the 2 methods in the *T*_2_ measurements above 95 ms were not adequate for radiotherapy planning (**Figure 4**). This result is well known in NMR, where it has been shown that *T*_2_ measurement is very sensitive to magnetic field heterogeneities (31). The use of SE sequences can partly correct this effect, but the gradients required in imaging have a significant effect on transverse relaxation. Our results are in agreement with the results of Kim et al. (18) at 3 T, but discordant with those of Lee et al. (10), who found a very good linear relationship between the 2 methods for *T*_2_ values between 30 and 110 ms.

Due to the measurement uncertainties of qMRI, reported by Nunez-Gonzalez et al. (2), and our final application for radiotherapy treatment planning for prostate and brain tissues (**Figure 4**), considering a quantitative accuracy below 20% in the *T*_1_ and *T*_2_ measurements seems to be sufficient. Indeed, Zavalla et al. (32) proposed a decision tree for the automatic segmentation–classification of prostate tissues in 3 T from *T*_1_ and *T*_2_ mappings. The application of this decision tree to our 5 patients who had pelvic scans was performed with the manually obtained VOIs of the different tissues on non-pathological areas (**Supplementary Table S3**). By considering that the relaxation times of *T*_1_ and *T*_2_ between 1.5 and 3 T were different (2, 33), our results showed correct classification of the prostate, fat, and muscle tissues regardless of the qMRI method. For other quantitative imaging modalities (positron emission tomography), a reproducibility error of 25%–30% is accepted when interpreting the response to treatment of cancerous lesions (34). In addition, Stikov et al. (14) showed that the variability of *T*_1_ measurements is about 10% higher *in vivo* than in the phantom.

The values measured with the MRI scanner were highly erroneous in relation to the theoretical values of relaxation times. The measurement accuracy of *T*_1_ and *T*_2_ degraded for high values of *T*_1_ and *T*_2_, showing that there is significant scope for MRI to improve the accuracy of relaxation time measurement. However, these underestimates of *T*_2_ measurements from theoretical values at 1.5 T of 500 ms can be explained by the choice of the TR value used for the ME-SE method, which does not allow correct sampling of the signal decay in *T*_2_. Additionally, to obtain correct *T*_2_ measurements with this sequence, it would be necessary to increase the value of TE, and thus TR, at the detriment of the acquisition time. From a methodological point of view, we chose very similar acquisition times between MAGiC^®^ and the conventional sequences for a relevant comparison. This duration corresponded to a clinically compatible acquisition time. For these same reasons, the GRE-VFA method was used for the conventional method instead of the IR method, which is acknowledged as the gold standard method for *T*_1_ measurement. The VFA method may overestimate the *T*_1_ values compared to IR (35) and affect the measurements if there is not a perfect destruction of the transverse magnetization before each flip angle; otherwise, the signal may deviate from the expected value as the flip angle increases. But this method has the advantage of speed and can be applied in 3D (14), which is interesting for a radiotherapy planning use. Recently, Nunez-Gonzalez et al. (2) have compared the MAGiC^®^ sequence to modified conventional methods based on a very short acquisition time (36). The results on phantoms were similar to ours, with 3 times faster acquisition time. However, this very promising sequence has to be evaluated under acquisition conditions dedicated to radiotherapy planning with an adapted voxel size and QAp analysis.

The results of our study showed the stability over time of qMRI measurement with MRI at 1.5 T for repeated measurements, which is a prerequisite for oncology follow-up. Our results suggest that a regular evaluation through quality control of the measurement of values over time is essential for such an application. The harmonization of this quality control on several MRI scanners would allow the comparison of the signal measurements in multicenter studies (37). Our results showed extreme values for *T*_1_ measurements obtained with both methods between 489.7 and 1,303.8 ms at the time of one of our 14 measurements. Although the values varied in the same direction and modified the contrast between tissues, without altering the quality of the delineation, we believe that the insertion of a calibration object in the field of view (FOV) at the time of the acquisition of a patient could quantify the possible drift and allow an immediate correction of the obtained values. Thus, a fast quality control in real time could be performed before releasing the patient and performing a rescan, if necessary.

The QAp results revealed a slice thickness slightly away from the expected optimal value for both methods. This result can be very influential, especially in brain metastases for stereotactic radiosurgery (38), but can be compensated by a registration on a treatment planning CT (39). The SNR of the conventional method was significantly higher than that of the MAGIC^®^ method for low-contrast detectability. It is known in MRI that the flip angle error due to imperfection of the slice profile and local changes in the *B*_1_ field is a source of bias for quantitative MR (40). It has been shown that, in the conventional method, the distribution of flip angles across the slice can induce slice profile distortions and a large excess of signal from the slice edges in subsequent RF pulses and that this could be ameliorated by discarding the subsequent RF views (41). Other promising acquisition methods are currently being investigated, which could improve these results (2, 42). Geometric distortion was only evaluated in 2D in this study, whereas 3D sequences are recommended for radiotherapy to more easily reconstruct in multiple planes and to limit the occurrence of 2D-related artifacts (22) because the MAGiC^®^ method is currently only available in 2D on MRI systems. Further evaluation of 3D distortion should be performed upon implementation in MRI machines. Furthermore, the distortion measurement would have been more complete by measuring it in all *x* and *y* directions in addition to the phantom diameter measurement, but the Magphan^®^ SMR170 version does not allow for this more accurate assessment.

In this work, quantitative MRI relaxometry techniques were evaluated for integration into radiotherapy treatment planning of the brain and prostate. We believe that this imaging technique could provide significant assistance to the radiation therapist in tumor contouring. For radiotherapy planning, quantitative MRI relaxometry allows the generation of several synthetic MRI contrasts that can distinguish several close contrast structures from a single acquisition (**Videos S1** and **S2**). It thus offers the double advantage of contouring assistance and workflow optimization because it can replace the acquisition of several sequences requiring a longer time. qMRI could also evaluate changes induced by irradiation delivered in radiotherapy, as recently shown with MRI imaging of myelin water by multicomponent *T*_2_ relaxometry (43). The MAGiC^®^ method was initially proposed for the study of the brain. The results given in **Figures 7B**, **9** and **Supplementary Table S3** showed that its use seems possible on the prostate. This result is interesting for prostate cancer, given that several studies (44, 45) have shown that *T*_2_ mapping has good diagnostic performance and could provide an indication of the aggressiveness of the disease. The preliminary results on five patients presented here are encouraging and seem more favorable than those on phantoms compared to reference samples (**Figure 3B**). This result is counterintuitive as it is expected in MRI that the results on patients are worse than those on phantoms. The differences between the *in vitro* and *in vivo* measurements may be due to the design of the homemade phantom. Indeed, as the vials used were 1 cm in diameter, the proton environment may have been more sensitive to local variations in *T*_1_ and *T*_2_ relaxation times. This type of variation was not present in the *in vivo* measurements. Regardless, these results on patients must be confirmed by a clinical study on a larger number of patients. The sample concentrations of the *T*_1_ and *T*_2_ values of the homemade phantom were <2,386 and <24.7 ms, respectively, whereas minimum concentrations of 4,627 ms for *T*_1_ and 6 ms for *T*_2_ were required for the completeness of the range of human biological tissues. However, only the CSF for *T*_1_ and the lung for *T*_2_ had relaxation times at 1.5 T in this range (30). We verified on phantom that it was not necessary to apply a *B*_1_ field uniformity correction from the GRE-VFA method. However, Stikov et al. (14) showed that the variability of the *T*_1_ measurements was affected by the *B*_1_ heterogeneity. However, it was not possible with our MRI system to parameterize the *B*_1_ *(* 25) measurement method used at a slice thickness less than 5 mm. Furthermore, this would have increased the global acquisition time of the conventional method. Errors in the absolute values of quantitative relaxometry MRI techniques may also arise from the software used to generate the *T*_1_ maps (14, 46, 47). But an analysis of the performance of the software generating the maps is beyond the scope of this work. The study of the two patient groups of brain and male pelvis only included a small population. These results need to be confirmed by a study on a larger sample of patients, and it would be interesting to study other applications for radiotherapy such as dose calculation and treatment simulation. In addition, we compared the relaxation time measurements between the two measurement methods by manually drawing VOIs in each anatomical area. Although we used average VOI values for our analyses, this method may have measurement variabilities between the different qMRI mappings obtained intra- and inter-patient.

## 5 Conclusions

Our results showed good agreement between the MAGiC^®^ method and the conventional sequences with respect to the reference values for *T*_1_ values between 500 and 1,304 ms and *T*_2_ values between 25 and 95 ms, with good reproducibility and repeatability at 1.5 T. Along with the QAp results, our work showed that it is now possible to use these imaging methods for anatomical segmentation for radiotherapy treatment planning. The results of the patient study are encouraging for the use of the MAGiC^®^ sequence with a BODY coil for prostate qMRI and should be confirmed in a study with a larger population.

## Data Availability Statement

The original contributions presented in the study are included in the article/**Supplementary Material**. Further inquiries can be directed to the corresponding author.

## Ethics Statement

The studies involving human participants were reviewed and approved by IRB Henri Becquerel Center. The patients/participants provided written informed consent to participate in this study. Written informed consent was obtained from the individual(s) for the publication of any potentially identifiable images or data included in this article.

## Author Contributions

PG, IG, and DG: conceived and designed this study. PG, AD, and DG: material preparation and data collection. PG and ET: statistical analysis. PG, SH, ST, PV, PH, and DG: visualization, manuscript writing, and submission. All authors contributed to the article and approved the submitted version.

## Conflict of Interest

A scientific collaboration between Henri Becquerel Center and Olea Medical^®^ allowed the use of OleaNova+^®^ software for this study.

The authors declare that the research was conducted in the absence of any commercial or financial relationships that could be construed as a potential conflict of interest.

## Publisher’s Note

All claims expressed in this article are solely those of the authors and do not necessarily represent those of their affiliated organizations, or those of the publisher, the editors and the reviewers. Any product that may be evaluated in this article, or claim that may be made by its manufacturer, is not guaranteed or endorsed by the publisher.

## Supplementary Material

Supplementary Video 1 MRI synthetic contrast obtained with the MAGiC^®^ sequence on a brain.

Supplementary Video 2 MRI synthetic contrast obtained with the MAGiC^®^ sequence on a prostate.
