# Supplementary material for: Synthetic MRI for Radiotherapy Planning for Brain and Prostate Cancers: Phantom Validation and Patient Evaluation
Source: Front Oncol. 2022 Apr 20;12:841761. doi: 10.3389/fonc.2022.841761 (PMC9065558; doi:10.3389/fonc.2022.841761)
Supplement: Supplementary file 1 [file DataSheet_1.docx]

Supplementary Material

**Figure S1.** B_1_map calibration curve HNU coil obtained by averaging homemade phantom measurement per week for six weeks. The mean relative differences are -1.6%.

**Figure S2.** B_1_map calibration curve BODY coil obtained by averaging homemade phantom measurement per week for six weeks. The mean relative differences are 1.8%.

|  |  | **MAGiC T1** | | | **GRE-VFA** | | |
| --- | --- | --- | --- | --- | --- | --- | --- |
| Reference values T1 (ms) | | Values measured (ms) | CV (%) | Relative differences (%) | Values measured (ms) | CV (%) | Relative differences (%) |
| Insert 1 | 29.1 | 500.0 | 0.0 | 1617.17 | 26.8 | 4.9 | -7.99 |
| Insert 2 | 57.7 | 707.0 | 127.0 | 1126.13 | 53.5 | 4.7 | -7.22 |
| Insert 3 | 113.1 | 301.0 | 0.7 | 166.15 | 109.1 | 4.5 | -3.53 |
| Insert 4 | 217.8 | 301.0 | 0.3 | 38.22 | 224.8 | 7.4 | 3.25 |
| Insert 5 | 489.7 | 518.0 | 4.1 | 5.78 | 568.5 | 7.5 | 16.09 |
| Insert 6 | 838.9 | 771.0 | 3.1 | -8.10 | 840.9 | 8.8 | 0.24 |
| Insert 7 | 1303.8 | 1323.0 | 4.2 | 1.47 | 1446.7 | 9.4 | 10.95 |
| Insert 8 | 2385.7 | 2819.0 | 10.5 | 18.16 | 2648.1 | 6.9 | 11.00 |
|  |  | **MAGiC T2** | | | **ME-SE** | | |
| Reference values T2 (ms) | | Values measured (ms) | CV (%) | Relative differences (%) | Values measured (ms) | CV (%) | Relative differences (%) |
| Insert 1 | 24.7 | 26.0 | 11.5 | 5.3 | 26.37 | 2.7 | 6.8 |
| Insert 2 | 48.8 | 48.0 | 4.2 | -1.6 | 51.4 | 3.2 | 5.5 |
| Insert 3 | 95.2 | 89.0 | 6.7 | -6.5 | 100.6 | 3.3 | 5.7 |
| Insert 4 | 181.7 | 157.0 | 7.0 | -13.6 | 182.2 | 6.1 | 0.2 |
| Insert 5 | 399.6 | 337.0 | 17.2 | -15.7 | 345.3 | 9.6 | -13.6 |
| Insert 6 | 665.6 | 472.0 | 19.1 | -29.1 | 453.8 | 21.7 | -31.8 |
| Insert 7 | 997.5 | 641.0 | 37.6 | -35.7 | 616.0 | 29.6 | -38.2 |
| Insert 8 | 1311.3 | 1092.0 | 55.3 | -16.7 | 778.4 | 72.4 | -86.1 |

**Table S1.** T_1_ and T_2_ measurements, associated CVs and the relative differences compared to the reference values for HNU coil at 1.5 T.

|  |  | **MAGiC T1** | | | **GRE-VFA** | | |
| --- | --- | --- | --- | --- | --- | --- | --- |
| Reference values T1 (ms) | | Values measured (ms) | CV (%) | Relative differences (%) | Values measured (ms) | CV (%) | Relative differences (%) |
| Insert 1 | 29.1 | 324.0 | 19.8 | 1012.7 | 33.11 | 5.7 | 13.8 |
| Insert 2 | 57.7 | 327.0 | 0.6 | 467.1 | 67.09 | 5.6 | 16.3 |
| Insert 3 | 113.1 | 301.0 | 0.7 | 166.2 | 132.42 | 4.2 | 17.1 |
| Insert 4 | 217.8 | 301.0 | 0.7 | 38.2 | 243.08 | 7.7 | 11.6 |
| Insert 5 | 489.7 | 528.0 | 6.8 | 7.8 | 535.98 | 8.3 | 9.5 |
| Insert 6 | 838.9 | 690.0 | 11.4 | -17.8 | 815.68 | 11.6 | -2.8 |
| Insert 7 | 1303.8 | 1092.0 | 16.6 | -16.2 | 1403.35 | 10.5 | 7.6 |
| Insert 8 | 2385.7 | 1987.0 | 41.9 | -16.7 | 2833.77 | 20.3 | 18.8 |
|  |  | **MAGiC T2** | | | **ME-SE** | | |
| Reference values T2 (ms) | | Values measured (ms) | CV (%) | Relative differences (%) | Values measured (ms) | CV (%) | Relative differences (%) |
| Insert 1 | 24.7 | 26.0 | 11.5 | 5.3 | 26.6 | 3.4 | 7.6 |
| Insert 2 | 48.8 | 43.0 | 9.3 | -11.8 | 51.6 | 3.7 | 5.7 |
| Insert 3 | 95.2 | 80.0 | 7.5 | -16.0 | 103.0 | 4.8 | 8.2 |
| Insert 4 | 181.7 | 139.0 | 23.7 | -23.5 | 188.8 | 7.2 | 3.9 |
| Insert 5 | 399.6 | 284.0 | 22.2 | -28.9 | 419.9 | 21.6 | 5.1 |
| Insert 6 | 665.6 | 388.0 | 58.0 | -41.7 | 460.3 | 24.5 | -30.8 |
| Insert 7 | 997.5 | 726.0 | 72.6 | -27.2 | 596.17 | 63.0 | -40.2 |
| Insert 8 | 1311.3 | 936.0 | 73.3 | -28.6 | 718.24 | 96.7 | -45.2 |

**Table S2.** T_1_ and T_2_ measurements, associated CVs, and the relative differences compared to the reference values for BODY Coil at 1.5 T.

**A**

| **HEAD Coil** | **MAGiC T1** | **GRE VFA** | **MAGiC T2** | **ME SE** |
| --- | --- | --- | --- | --- |
| White Matter | 686 | 762 | 92 | 81 |
|  | 713 | 788 | 99 | 90 |
|  | 904 | 910 | 84 | 87 |
|  | 844 | 827 | 87 | 86 |
|  | 980 | 939 | 99 | 87 |
| Grey Matter | 1308 | 1450 | 98 | 101 |
|  | 1287 | 1367 | 101 | 104 |
|  | 1235 | 1388 | 111 | 109 |
|  | 1055 | 1169 | 99 | 98 |
|  | 1205 | 1193 | 118 | 113 |
| Cerebral Spinal Fluid | 4194 | 4621 | 1142 | 1059 |
|  | 4292 | 4655 | 1633 | 1060 |
|  | 4296 | 4958 | 1502 | 1402 |
|  | 3584 | 4365 | 1007 | 1007 |
|  | 4291 | 4333 | 1654 | 1389 |

**B**

| **BODY coil** | **MAGiC T1** | **GRE VFA** | **MAGiC T2** | **ME SE** |
| --- | --- | --- | --- | --- |
| Fat | 315 | 291 | 144 | 137 |
|  | 335 | 280 | 128 | 119 |
|  | 311 | 292 | 136 | 126 |
|  | 326 | 279 | 137 | 129 |
|  | 328 | 302 | 131 | 123 |
| Muscle | 859 | 971 | 49 | 46 |
|  | 907 | 941 | 63 | 62 |
|  | 759 | 983 | 50 | 49 |
|  | 818 | 970 | 53 | 49 |
|  | 814 | 949 | 53 | 48 |
| Prostate | 1599 | 1696 | 117 | 106 |
|  | 1433 | 1346 | 92 | 88 |
|  | 1213 | 1430 | 91 | 89 |
|  | 1259 | 1267 | 89 | 86 |
|  | 1122 | 1272 | 89 | 84 |

**Table S3.** Results of measurements of T_1_ and T_2_ with the MAGIC® and GRE-VFA and ME-SE sequences of (A) the five patients brain (HNU coil) and (B) five patients pelvis (BODY coil).
